# Nutraceutical Approach to Preventing Coronavirus Disease 2019 and Related Complications

**DOI:** 10.3389/fimmu.2021.582556

**Published:** 2021-06-28

**Authors:** Giuseppe Derosa, Pamela Maffioli, Angela D’Angelo, Francesco Di Pierro

**Affiliations:** ^1^ Department of Internal Medicine and Therapeutics, University of Pavia, Pavia, Italy; ^2^ Laboratory of Molecular Medicine, University of Pavia, Pavia, Italy; ^3^ Velleja Research S.r.l., Milan, Italy; ^4^ Digestive Endoscopy & Gastroenterology, Poliambulanza Hospital, Brescia, Italy

**Keywords:** SARS-CoV-2, COVID-19, infectious diseases, nutraceuticals, inflammation

## Abstract

**Introduction:**

Several months ago, Chinese authorities identified an atypical pneumonia in Wuhan city, province of Hubei (China) caused by a novel coronavirus (2019-nCoV or SARS-CoV-2). The WHO announced this new disease was to be known as “COVID-19”.

**Evidence Acquisition:**

Several approaches are currently underway for the treatment of this disease, but a specific cure remains to be established.

**Evidence Synthesis:**

This review will describe how the use of selected nutraceuticals could be helpful, in addition to pharmacological therapy, in preventing some COVID-19-related complications in infected patients.

**Conclusions:**

Even if a specific and effective cure for COVID-19 still has some way to go, selected nutraceuticals could be helpful, in addition to pharmacological therapy, in preventing some COVID-19-related complications in infected patients.

## Introduction

Several months ago, Chinese authorities identified an atypical pneumonia in Wuhan city, province of Hubei (China) and they reported it to the China Country Office of the World Health Organization (WHO) on 31^st^ December 2019. On 7^th^ January 2020, Chinese authorities isolated the aetiological agent as a novel coronavirus (2019-nCoV). The genetic sequence of 2019-nCoV was shared with other countries to develop specific diagnostic kits on 12^th^ January 2020. In the following days, the Thai Ministry of Public Health, the Japanese Ministry of Health, Labour and Welfare, and the Korean Ministry of Health, Labour and Welfare reported their first imported case of laboratory-confirmed 2019-nCoV on 13^th^ January, 15^th^ January, and 20^th^ January 2020, respectively. The International Committee on Taxonomy of Viruses classified the agent as SARS-CoV-2, given its similarity with the symptoms induced by SARS-CoV-1.

On 22^nd^ January 2020 the virus appeared in Europe, in Bavaria (Germany) from a German patient infected by an individual from Shanghai (China); on 25^th^ January 2020, the virus was identified in Italy, in the area of ​​Basso Lodigiano.

On 30^th^ January 2020, the WHO declared the situation a public health emergency (1) and on 11^th^ February 2020, the WHO announced that the new disease was to be known as “COVID-19”, an acronym of “COronaVIrus Disease-2019”.

On 20^th^ February 2020, Italian Authorities identified the first symptomatic COVID-19 patient at the Emergency Department of Codogno Hospital, Basso Lodigiano, province of Lodi.

On 26^th^ February 2020, the Director‐General of the WHO announced that the number of new cases of COVID‐19 reported outside China had exceeded the number of new cases in China (2). On 11^th^ March 2020, the WHO declared this viral disease as pandemic. Cases subsequently increased exponentially, reaching a total of 4.044.762, with 121.177 deaths, in Italy on 9^th^ May 2021. As of 11^th^ May 2021, the WHO has reported 158.551.526 cases and 3.296.855 deaths that have occurred worldwide (3).

## Aetiology

Coronaviruses (CoVs) include a large group of single-stranded RNA viruses, with a crown-like appearance under an electron microscope.

CoVs were identified in the mid-1960s and they can infect humans and some animals (including birds and mammals). Their primary targets are the epithelial cells of the respiratory and gastrointestinal tracts.

The subfamily Orthocoronavirinae of the Coronaviridae family is classified into four CoV genera: alpha-CoV, beta-CoV, delta-CoV, and gamma-CoV. The beta-CoV genus is further divided into five subgenera (including Sarbecovirus) (4).

To date, seven CoVs are capable of infecting humans: 1) common human CoVs: HCoV-OC43 and HCoV-HKU1 (beta-CoV), and HCoV-229E and HCoV-NL63 (alpha-CoV) - they are responsible of common colds, but also of serious lower respiratory tract infections; and 2) other human CoVs (beta-CoV): SARS-CoV-1, MERS-CoV, and SARS-CoV-2 (5).

SARS-CoV-2 is a single-stranded, positive-sense RNA virus, with a diameter of 60-140 nm with a round or elliptic shape. It shares 96% sequence identity with bat genome (bat-SL-CoVZC45), suggesting a very recent host shift into humans (4, 5).

## Transmission

CoVs are hosted in nature by bats and the many CoVs present in humans are thought to derive from those in bats, which represent a reservoir (6).

Studies on the genomic sequence of SARS-CoV-2 identified a similarity of 50, 79, and 96% to MERS, SARS, and bat SARS-related CoV, respectively (7).

Currently, the specific route of transmission from natural reservoirs is unknown, but some studies suggest that the pangolin, which has viruses with more than 99% of the genome identical to SARS-CoV-2, is a worthy candidate for transmission (8).

The new CoV, SARS-CoV-2 is a respiratory virus spreading through the breath droplets of infected individuals. SARS-CoV-2 spreads through: 1) saliva, coughing, and sneezing; 2) direct personal contact; and 3) hands, for example, by touching the mouth, nose, or eyes with contaminated (unwashed) hands. Similarly to SARS-CoV-1, SARS-CoV-2 uses the angiotensin-converting enzyme II (ACE2) receptor expressed by human cells as an entry receptor (9).

The virus can survive on different surfaces for days and remain viable in aerosols for hours (10). Asymptomatic patients can carry and spread the virus, a high viral load has been identified in pharyngeal samples from minimally symptomatic patients during the initial period of the disease. The highest transmission rates correlate with disease severity and they are particularly pronounced in hospital settings, just as for SARS and MERS. The incubation period is between 3 and 14 days, and the onset of symptoms has been reported up to 14 days after exposure (11), providing the basis for the recommended length of quarantine/self-isolation (12).

## Clinical Patterns

The symptoms that patients with COVID-19 may exhibit are variable. In the period from November to December 2019 in Italy, many general practitioners visited patients with a symptomatology that resembled influenza and which was not a common cold.

The symptoms, however, were similar and therefore confusing, such as fever, cough, and myalgias. Sometimes, nausea and diarrhoea could precede fever and respiratory symptoms. The persistence of this symptomatology, atypical for being a complicated influenza, caused doctors to request chest X-rays, with images often indicating pneumonia. After the statements from the Chinese authorities, the picture became clearer. Generally, patients are characterised by three main symptoms: 1) fever, 2) dry cough, and 3) dyspnoea.

In some patients, the latter may not be present initially, as this symptom may be decisive for hospitalisation or home follow-up. We have since learned that if COVID-19 is suspected and hospitalisation of the patient is envisaged, the emergency room must be avoided and testing and management must be conducted in rapidly accessible areas with a low risk of exposure to mitigate spread and to avoid overwhelming the healthcare system.

A first test to verify that the patient has been infected is swab sampling from the oropharynx or nasopharynx.

We can classify the infection as symptomatic or asymptomatic. Prodromal signs of the infection include conjunctivitis (often the first sign) or gastrointestinal symptoms such as nausea, vomiting, abdominal pain, and diarrhoea.

Some patients have only abdominal pain, anorexia, and dyspnoea without fever. Anosmia and dysgeusia can be present (13).

Older age, male sex, and smoking worsen the prognosis such as the presence of associated pathologies including obesity, hypertension, diabetes mellitus, cardiovascular disease, chronic kidney disease, and respiratory diseases. The greater is the number of risk factors and associated pathologies, the higher is the risk of a poor prognosis (14).

A mild symptomatology can resolve without particular medical treatment at home or it can progress towards bilateral interstitial pneumonia and respiratory failure, requiring hospitalisation. Thrombotic complications seem to emerge as an important issue in infected patients. Clinical parameters to check include the presence of leucocytosis, thrombocytopenia, an elevated prothrombin time and partial thromboplastin time, and elevated levels of fibrinogen and D-dimers (15).

Patients can rapidly progress towards acute respiratory distress syndrome (ARDS) with multiple organ dysfunction syndrome (MODS) and death (16).

## Therapy

COVID-19 therapy is only supportive and prevention is the only way to reduce transmission and limit its spreading. Despite social distancing, hygiene measures, and screening, the COVID-19 pandemic is spreading quickly all over the world, with the risk that health systems will be unable to withstand its impact. At first, treatment with lopinavir/ritonavir and chloroquine/hydroxychloroquine were attempted (17). In addition, a large number of antibiotics have been used, including ceftriaxone with azithromycin or piperacillin/tazobactam and doxycycline, and azithromycin alone.

From mid-March 2020, lopinavir/ritonavir was replaced with remdesivir and tocilizumab, also heparin was suggested as a potential treatment. Regarding glucocorticoids and their beneficial effects in the early stages of the disease, data are not so clear (18).

The use of plasma with antibodies obtained from patients recovered from COVID-19 seems to have a good rationale, although data are still from a limited number of patients (19).

While the development of effective “old-new” and new drug therapies is proceeding, with little success, vaccines are now available. There are several vaccines available. The first mass vaccination programme started in early December 2020. World Health Organization issued an Emergency Use Listing (EULs) for the Pfizer COVID-19 vaccine (BNT162b2) on December 31^st^ 2020. On February 15^th^ 2021, WHO issued EULs for two versions of the AstraZeneca/Oxford COVID-19 vaccine, manufactured by the Serum Institute of India and SKBio. On March 12^th^ 2021, WHO issued an EUL for the COVID-19 vaccine Ad26.COV2.S, developed by Janssen (Johnson & Johnson). WHO is on track to EUL other vaccine products through June. The most common side effect reported is soreness at the injection site. Less frequent side effects include fatigue, headache, muscle aches, chills, joint pain, and possibly some fever.

On this basis, this review will describe how the use of selected nutraceuticals could be helpful, in addition to pharmacological therapy, in preventing some COVID-19-related complications in infected patients.

References for this review were identified through searches within PubMed for articles published from January 2001 to May 2021 using the terms “Antiviral substances”, “Nutraceuticals”, and “COVID-19”. Articles resulting from these searches and relevant references cited in those articles were reviewed. Only articles published in English were included.

### Nutraceuticals Possibly Helpful in Treating COVID-19-Infected Patients

Since ancient time vegetables have been used by mankind as an important source of medicines. Currently, a wide portion of the world population still rely on botanical remedies to meet their health needs. Now we are witnessing an increasing interest and use of therapies with botanicals used to formulate healthcare products. Just to say, U.S. sales of herbals have increased by 8.5% from 2016 to 2017, recently reaching a likely estimated total of about 10 billion USD per year. Clearly, the increased public interest in botanicals has stimulated a greater scientific awareness aimed to better understand the pharmacological activity of medicinal plants. As it is well-known, many actives obtained from plant sources are indeed described to be endowed with pharmacological activities, and historically vegetables have yielded many important drugs for human use (i. e. morphine, vinblastine, vincristine, paclitaxel, and artemisinin). Although Big Pharma is not focusing on developing natural drugs from botanicals, natural products remain an important and viable source of lead compounds in many drug research programs. Finally, the development of natural products for the prevention and/or treatment of diseases continue to attract worldwide medical and financial attention (20).

As regards to the current coronavirus pandemic, several nutraceutical actives have indeed been described as being protective against some of those symptoms that are also considered manifestations of COVID-19 in infected patients. These include probiotics, polyphenols, high molecular weight polysaccharides, lectins, berberine, and others. Their use could therefore be possibly considered, if not in a curative context, then at least as a prophylactic, capable of reducing the burden and severity of the disease.

### Polyphenols

From a pharmacological perspective, the most important targets with respect to SARS-CoV-2 include 3-chymotrypsin-like protease (3CLpro), papain-like protease (PLpro), RNA-dependent RNA polymerase, and spike (S) protein (21). The S protein interacts directly with human ACE2, allowing the virus to enter cells. Naturally occurring antiviral agents acting against general CoVs were briefly reviewed six years ago (22), while more recent reviews by Pang et al. (23) and Lu (24) on therapies for COVID-19 made only short mention of natural therapeutics and did not explore the individual active compounds. In light of the current COVID-19 pandemic, some more precise information on extracts and/or individual compounds derived from natural products which show potential antiviral bioactivity for the inhibition of CoV can be given. Unarguably, the botanical family of compounds that demonstrates the greatest antiviral activity is the polyphenols. Quercetin showed an IC50 of 8.6 ± 3.2 μM against SARS-CoV-1 PLpro (25). Quercetin is a common flavonoid found in many foods, berries, and herbs (and therefore, in extracts). Bearing in mind its very poor pharmacokinetic profile in animals and in humans, a bioavailable form of quercetin could be considered a better option to test (26). The structurally similar polyphenolics myricetin (Opuntia ficus-indica) and scutellarein (Scutellaria baicalensis) display reasonable levels of inhibitory activity against SARS-CoV-1 helicase (27). Testing of a multi-fractionated ethanolic extract obtained from Psoralea corylifolia seeds has also identified polyphenols mediating activity versus SARS-CoV-1 PLpro (28). Furthermore, six phenolic phytochemicals were isolated from other ethanolic extracts and identified as bavachinin, neobavaisoflavone, isobavachalcone, 4′-O-methylbavachalcone, psoralidin, and corylifol A; all showed antiviral activity that varied widely, with IC50 values between 4.2 and 38.4 μM (28).

### Lectins

Botanical lectins, proteins binding specifically and reversibly to carbohydrate groups (29), may inhibit SARS-CoV-2. They showed some effects as antiviral agents against viruses such as influenza and herpes simplex (30), as well as Ebola (31). Of note, high plasma levels of recombinant human mannose-binding lectin in mice allowed them to survive otherwise fatal Ebola infections (32). Screening the activity of a broad range of 33 plant lectins against SARS-CoV-1 using a cytopathic effect inhibition assay, EC50 values as low as 0.45 ± 0.08 μg/mL for Lycoris radiata agglutinin were found (33). A direct action at the stage of viral attachment or the end of the infectious viral cycle was believed to be the most likely underlying mechanism. Clinically, other lectins are well tolerated (34). Thus, lectins could be one of the more promising classes of naturally derived compounds for the treatment of SARS-CoV-2 infections.

### High Molecular Weight Polysaccharides

In recent years, several natural products, mainly from herbal and mushroom extracts; for instance, Echinacea spp., ginseng, Astragalus (35), or shiitake derivatives (36), have been investigated for their immunomodulatory role against infective, including respiratory, diseases. Their use is generally considered a form of complementary and/or traditional medicine. For these natural extracts, the active compounds are thought to be high molecular weight polysaccharides, which have beneficial effects on health and disease probably due to a direct role in immunity (37). Regarding fungal derivatives, some mushroom extracts are used worldwide, mainly as dietary supplements and functional foods, to treat pathologies associated with immune dysregulation, including respiratory infections (38). The effects of selected mushroom derivatives on the immune system are likely due to the presence of β-glucans, which are thought to be capable of affecting innate and adaptive immune responses (39). In addition, other studies have reported the activation of NK and/or T cells by a different type of high molecular weight polysaccharides, named α-glucans, effective in human and animal immunity, therefore suggesting a theoretical role in defending the host against respiratory infections (40). The most investigated and well-documented α-glucan formulation, active hexose correlated compound (AHCC), has been shown to be effective against different types of infectious diseases caused by viruses such as West Nile virus, influenza virus, avian influenza virus, hepatitis C virus, and human papillomavirus (41–47). In those studies, it was shown that NK cells, natural killer T (NKT) cells, and gamma delta T (γδ T) cells are modulated and activated by AHCC intake. NK, NKT, and γδ T cells represent primary interferon-gamma (IFN-γ) producers (48–50). As is well known, low pathogenic human CoV infects the upper airways and causes seasonal mild to moderate cold-like respiratory illnesses in healthy individuals. In contrast, the highly pathogenic viruses infect the lower respiratory tract and cause severe pneumonia, which sometimes leads to fatal acute lung injury (ALI) and ARDS, resulting in high morbidity and mortality (51). From this perspective, the host IFN-γ response is, among others, one of the factors considered likely to be protective in preventing ALI and ARDS in subjects infected with highly pathogenic CoV (52). Although AHCC has never been tested against human CoVs, its mechanism of action makes it one of the best possible candidates to promote immunity in those subjects not yet infected but at considerable risk, or in those recently infected by SARS-CoV-2 (53). The most thoroughly investigated herbal derivatives in terms of their immunological properties are probably extracts from Echinacea angustifolia, Echinacea pallida, and Echinacea purpurea (54). Many human trials and meta-analyses have described their role in immunity and their capability to protect the host from URTI (55). The most highly purified and chemically characterised Echinacea extract is probably Polinacea^®^. This has been described as promoting an interferon-mediated, T-cell response against viruses both in animals and in humans, with its mechanism of action also consisting of non-concomitant production of a proinflammatory cytokine cascade supported by IL-6 and/or TNF-α release (56–59). This unique feature makes the extract a good candidate for COVID-19 prevention and possibly treatment.

### Natto

Natto is a cheese-like food composed of soybeans fermented with Bacillus subtilis. It is a traditional food in Asian countries for more than 2,000 years and it plays a role in long life within the Japanese population. Several studies reported that high levels of natto consumption reduce the risk of total cardiovascular disease mortality and, in particular, reduce risk of mortality from ischaemic heart diseases (60) Relatively little was known regarding the mechanism by which natto intake led to an overall improvement in cardiovascular health until the 1980s. A potent fibrinolytic enzyme called nattokinase was discovered in natto in 1987 (61), and since then, nattokinase has been the subject of a considerable amount of research conducted in both Asian countries (Japan, China, Korea) and the United States. These studies have established that this enzyme, a 275-amino acid alkaline protease with a molecular weight of approximately 28 kDa, comprises the most active ingredient of natto, mediating its positive effects on cardiovascular health. Not only does nattokinase possess potent fibrinolytic/antithrombotic activity (62–64), but nattokinase has also been shown to exert antihypertensive, anti-atherosclerotic, lipid-lowering, antiplatelet/anticoagulant, and neuroprotective effects in both animal and human studies (65–72). All of these pharmacologic actions of nattokinase are relevant to the prevention and treatment of COVID-19. For example, supplementation with nattokinase enhanced markers of fibrinolysis and anticoagulation in human subjects, along with decreasing blood pressure and atherosclerosis (73–75). The most unique characteristic of nattokinase is that it has multiple cardiovascular disease-related preventative and alleviating pharmacologic effects (as mentioned, it has antithrombotic, antihypertensive, anticoagulant, anti-atherosclerotic, and neuroprotective effects): to the best of our knowledge, there are no other drugs with a similar range of pharmacologic properties. In addition, nattokinase is a natural product considered to be a “nutraceutical” in many countries throughout the world; it can be administered orally; it has a proven safety profile; it is inexpensive to use; and it provides many advantages in comparison to other pharmaceutical products. It therefore has the potential to be developed as a new-generation drug for the prevention, treatment, and long-term care of cardiovascular disease and, perhaps, COVID-19.

### Berberine

Berberine, a quaternary ammonium salt from the protoberberine group of isoquinoline alkaloids, is mainly noted for its anti-lipogenic and hypoglycaemic effects (76–81). There is also evidence that berberine hydrochloride has antibacterial, antifungal, antiprotozoal, antihelminthic, and antiviral properties (82). Focusing on the antiviral activity of berberine, several studies assessed its antiviral effects against specific viruses such as influenza, cytomegalovirus (CMV), and herpes simplex. In *in vivo* studies, berberine administered to mice with influenza decreased mortality from 90% to 55%, reducing virus titres in the lungs on day 2 post-infection. The production of nitric oxide (NO) and inducible nitric oxide synthase (iNOS) was repressed, along with inhibition of the transcription and expression of TNF-α and monocyte chemoattractant protein-1 (MCP-1). The possible mechanism underlying the therapeutic effects of berberine on influenza-induced viral pneumonia may be the inhibition of viral infection, as well as the improvement of pathogenic changes by repressing inflammatory substance release (83). Regarding its action in herpes simplex virus infection, berberine may interfere with the viral replication cycle after viral penetration and no later than the viral DNA synthesis step (84). Berberine also seems to have anti-human CMV (HCMV) activity, interfering with intracellular events after viral penetration into the host cells and before viral DNA synthesis (85).

### Botanicals Endowed With Anti-Inflammatory Properties

One of the characteristics more thoroughly described for nutraceuticals, especially those derived from herbs, is that of being anti-inflammatory. Due to the large number of these derivatives, their complete and exhaustive description would go far beyond the remit of this review. However, if we limit our investigation to those extracted derivatives that have been extensively documented, including those used clinically and those for whom molecular details of the active ingredients contained within are precisely known, the list is considerably reduced in number. Among those of particular interest, as they are likely to have an anti-inflammatory role in the second phase of SARS-CoV-2 infection, the phase represented by the well-known cytokine cascade which very often determines whether the patient will survive, we can list the derivatives from Curcuma longa, Boswellia serrata, and Nigella sativa. Curcuminoids, curcumin, and curcumin-like substances are polyphenolic structures that do not demonstrate direct antiviral activity (and thus, they have not been listed in the earlier subsection dedicated to polyphenols, 3.2) but they have been described in terms of their antagonistic capacity towards IL-6, IL-1, and TNF-α (86). As for quercetin, curcuminoids also appear to be characterised by a low oral bioavailability. For this reason, their co-administration with adjuvants such as piperine or the formation of complexes with phospholipids may be valuable (87, 88). The highly standardised extract containing boswellic acids and obtained from the resin of Boswellia serrata has also been widely described for its unique antagonistic action against the inflammatory cytokine (IL-6, TNF-α) cascade (89). Even boswellic acids, unless used for their intestinal anti-inflammatory properties, are more clinically active if complexed with phospholipids as they too are characterised by a suboptimal, if not poor, pharmacokinetic profile in animals and humans (90). Finally, we come to Nigella sativa. This is defined by the presence of thymoquinone, an active ingredient known for its lipid-lowering, antidiabetic (91), and, above all, anti-inflammatory actions (92–94). Being a herbal derivative in an oily form, there are no major limitations in terms of pharmacokinetics associated with thymoquinone and it could therefore be an excellent and easily administered nutraceutical candidate capable of counteracting the cytokine storm that characterizes SARS-CoV-2 infection.

### Lactoferrin

Lactoferrin is a highly conserved, iron-binding 80-kDa glycoprotein that is expressed and secreted by glandular cells and is found in most body fluids including bovine and human milk. Lactoferrin seems to be able to moderate the host response to infections both stimulating the immune system to counteract germ invasion and preventing harmful host immune and inflammatory responses (95).

Starting from the eighties the list of lactoferrin-susceptible, both naked and enveloped as well as DNA and RNA, human viruses has grown and to date includes cytomegalovirus, herpes simplex virus, human immunodeficiency virus (HIV), rotavirus, poliovirus, respiratory syncytial virus, hepatitis B virus, hepatitis C virus (HCV), parainfluenza virus, alphavirus, hantavirus, human papillomavirus, adenovirus, enterovirus 71, echovirus 6, influenza A virus and Japanese encephalitis virus (96, 97).

Particularly relevant is the lactoferrin ability to inhibit pseudo-typed SARS-CoV (the human coronavirus that is most closely related to SARS-CoV-2, which causes COVID-19) with a 50% inhibitory concentration of 0.7 μM (98).

Human oral supplementation (100-1000 mg/day) of lactoferrin against pathologic viruses was found to reduce the incidence of colds and cold-like symptoms as well as to ameliorate rotaviral gastroenteritis (95). Moreover, a randomised controlled study involving 111 HCV patients reported a significant decrease in HCV viral titres and sustained virological response in the lactoferrin group (99).

Besides to possibly show antiviral effects, lactoferrin has immunomodulatory and anti-inflammatory actions. Lactoferrin has the unique potential to maintain immune and physiological homeostasis and to limit tissue damage by modulation of cytokines, chemokines and cell surface receptors involved in cascades of signalling pathways (99).

With regards to the possible anti-inflammatory role played by lactoferrin, its use reduced many biological reactions normally seen upon experimentally lipopolysaccharide administration in a dose-dependent manner, and in an earlier study a single dose of lactoferrin prior to lipopolysaccharide injection reduced the mortality of mice to 16.7% compared with 83.3% in controls (100).

Due to the promising results got in experimental studies, lactoferrin has been investigated in some clinical studies against sepsis and recently, a meta-analysis of well-performed randomised controlled trials involving about 4000 infant showed that lactoferrin reduces late-onset sepsis (101)⁠.

To date, no clinical data have been produced to confirm a possible anti-COVID-19 effect of lactoferrin. Anyway, lactoferrin strongly affects immune response and cellular inflammation. Therefore, this natural component could provide a promising effect in preventing respiratory infections and potentially also for COVID-19. If the assumption that lactoferrin could modulate an overactive immune and inflammatory response to viral infection is correct, lactoferrin could be a candidate adjunct treatment for severe cases of COVID-19 (102).

### Vitamins, Minerals, and Omega-3 Fatty Acids

Since COVID-19 dysregulates the immune system, it seems important to evaluate the role played on immunity by simple nutritional elements like vitamins, minerals, and fomega-3 fatty acids.

Vitamin D has a well-known impact on immunity. It enhances innate cellular immunity through stimulation of expression of anti-microbial peptides which also maintain tight and gap junctions and enhance the expression of anti-oxidative genes. Vitamin D also promotes the differentiation of monocytes to macrophages, whilst increasing superoxide production, phagocytosis and bacterial destruction. Moreover, vitamin D modulates the adaptive immune response, it suppresses T helper type-1 (Th1) cell function and decreases the production of pro-inflammatory cytokines IL-2 and interferon-gamma (INF-γ). Vitamin D also promotes anti-inflammatory cytokines by Th2 cells and indirectly suppressing Th1 cells diverting pro-inflammatory cells to an anti-inflammatory phenotype as well as stimulating suppressive regulatory T cells (103). Low plasma levels of vitamin D seem to increase incidence and severity of COVID-19 infection. COVID-19 patients had lower levels of vitamin D, with mean plasma concentrations half that of controls (104). This could suggest for vitamin D a role in boosting immunity against COVID-19 and in reducing human mortality. Of course, this hypothesis needs to be tested in severely controlled human trials.

Vitamin C seems to have protective actions in infectious disease. Vitamin C supplementation supports respiratory defence mechanisms, prevents viral infections, and reduces their duration and severity. Vitamin C also has anti-histamine properties that can improve flu-like symptoms. A number of important reviews have been devoted to clarify a possible anti-COVID-19 vitamin C role and our review could not go deeper being not dedicated exclusively to it (105). On the basis of the many data available, vitamin C supplementation could be surely considered a sensible option in micronutrient deficient individuals that are at risk of COVID-19 infection to assist with the prevention and support of immune responses. To this end, several clinical trials are evaluating Vitamin C supplementation in COVID-19 patients.

Zinc has immunomodulatory and anti-viral properties (106) which could be helpful in treating COVID-19 patients (107). Zinc supplementation can decrease COVID-19 related symptoms such as lower respiratory tract infection. These effects are likely due to inhibition of viral uncoating, binding and replication, and therefore may be relevant to COVID-19. Due to that, a clinical trial registered in Australia will be soon able to determine if the use of intravenous zinc administration in COVID-19 positive individuals could modify the course of the infection (108).

Eicosapentaenoic and docosahexaenoic fatty acids are polyunsaturated fatty acids (omega-3) with favourable effects on immunity and inflammation. Omega-3 fatty acids seems also to exert an anti-viral effects in case of influenza virus. Noteworthy, the use of omega-3 fatty acids improved oxygenation in COVID-19 patients, although real evidence is still missing (109). In our opinion anyway, the use of the omega-3 in COVID-19 patients should be done with caution, due to a counter-intuitive increase in oxidative stress and inflammation due to increased susceptibility of cellular membranes to damage (110). Therefore, until there are validated trial data, omega-3 supplementation, particularly in high doses, must be performed with care in this population.

Vitamin E, vitamin A, magnesium, and trace element selenium, have been also cited by researchers as possible tools to be used to counteract the impact of coronavirus on immunity, especially as regards to vulnerable subjects, including elderly and immunocompromised (111). Anyway, the findings available are scares and further evidence are required.

### Nutraceuticals for COVID-19?

There are limited proven therapeutic options for the prevention and treatment of COVID-19. The role of nutraceuticals, botanicals, vitamin and mineral approach “to feed immunity” has been explored by many researchers all over the world and currently there are several hypotheses to support their routine use. The aim of this narrative review was to investigate if nutraceutical supplementation could be beneficial in COVID-19. Although there are currently no published controlled clinical trials, also due to the novelty of SARS-CoV-2 infection, there is strong pathophysiologic rationale for exploring the use of nutraceuticals in this global pandemic, supported by many reports from international groups. On the other side, we cannot claim a role for nutraceutical that remains to be demonstrated, underlining the need not to make promises that would risk to be disappointed by future results.

## Conclusions

Even if a specific and effective cure for COVID-19 still has some way to go, selected nutraceuticals could be helpful, in addition to pharmacological therapy, in preventing some COVID-19-related complications in infected patients.

## Author Contributions

All authors were equally involved in writing this review. All authors contributed to the article and approved the submitted version.

## Conflict of Interest

Author FP was employed by the company Velleja Research S.r.l., Milan, Italy.

The remaining authors declare that the research was conducted in the absence of any commercial or financial relationships that could be construed as a potential conflict of interest.
